# Role of 24-hour Helpline Service in the Management of Diabetes During the Holy Month of Ramadan

**DOI:** 10.7759/cureus.7320

**Published:** 2020-03-18

**Authors:** Muhammad Saif Ulhaque, Awn Bin Zafar, Farrukh Ahmed, Muhammad Yakoob Ahmedani

**Affiliations:** 1 Medicine, Baqai Institute of Diabetology and Endocrinology, Baqai Medical University, Karachi, PAK; 2 Diet and Education, Baqai Institute of Diabetology and Endocrinology, Baqai Medical University, Karachi, PAK

**Keywords:** 24-hours telephonic helpline service, type 1 diabetes, type 2 diabetes, education, ramadan

## Abstract

Objective

To assess the role of the 24-hour helpline service in the management of diabetes during the holy month of Ramadan.

Methodology

This prospective study was conducted at the Baqai Institute of Diabetology & Endocrinology, Baqai Medical University, Karachi, Pakistan, between December 2017 and August 2018. Patients with type 1 and type 2 diabetes who were willing to observe the fast in Ramadan 2018 were included. Pre-Ramadan education on a one-to-one basis was given and the 24-hour helpline number was provided to each patient. Details of baseline characteristics, anthropometric measurements, and biochemical parameters (lipid profile, serum creatinine, urine D/R (protein), and glycated hemoglobin A1c pre- and post-Ramadan) were noted. Self-monitoring blood glucose (SMBG) recording books were provided to each patient to record blood sugar levels during Ramadan.

Results

A total of 102 patients, 41 (40.2%) with type 1 diabetes and 61 (59.8%) with type 2 diabetes were included. The mean age of patients with type 1 diabetes was 17.02±5.22 years and that of patients with type 2 diabetes was 49.48±9.68 years. Most of the patients were male in both groups. By using the 24-hour helpline service, 912 calls were made to patients, 502 calls for type 1, and 410 calls for type 2 diabetes. Mean HbA1c (%) level before and after Ramadan was 8.94±1.72 and 8.70±2.03 in patients with type 1 diabetes and 8.59±1.73 and 7.71±1.27 in patients with type 2 diabetes, respectively. In the last 10 days of Ramadan, the decreasing trends of mean self-monitoring of blood glucose (SMBG) in patients with type 1 diabetes was observed for pre-dawn meal (suhoor), pre-sunset meal (iftar), and before sleeping while this trend in type 2 diabetes was found in the pre-dawn meal (suhoor)and two hours after iftar.

Conclusion

Pre-Ramadan diabetes education and treatment adjustment by using a station-based, 24-hour helpline service during the holy month of Ramadan have a significant role in controlling blood sugar levels, thus minimizing the complications of diabetes, facilitating timely adjustments in treatment, and reducing unnecessary hospital visits and admissions.

## Introduction

Diabetes is one of the most alarming public health issues, especially for lower-middle-income countries [1]. In Pakistan, 7.5 million people have diabetes as reported by the International Diabetes Federation (IDF) atlas [2]. A recent second National Diabetes Survey of Pakistan (NDSP 2016-2017) has estimated that the prevalence of diabetes is 26.3% [3].

Fasting (abstaining from food and drink from dawn to sunset) during the month of Ramadan is one of the five main pillars of Islam [4]. Most Muslims are passionate about fasting during this month, although sick people are exempted. However, many diabetics may not perceive themselves as sick and a high proportion fasts [5-6]. A large epidemiology study of Muslims with diabetes in 13 Islamic countries - the Epidemiology of Diabetes and Ramadan (EPIDAR) study - showed 43% of patients with type 1 and 79% of those with type 2 diabetes fasting during Ramadan [7].

Patients with diabetes who intend to fast should have proper knowledge to fast and receive counseling about change in medication timings and dose, dietary changes, and the role of SMBG in order to avoid developing acute complications [8]. Diabetes is a condition that requires constant monitoring and adjustments of medication, resulting in frequent visits to the clinic. To minimize these visits and provide ongoing support, a system is available in the form of the 24-hour helpline service. This is an easily accessible and low-cost medium, which is considered an effective alternative [9]. If prompt advice is given, the frequency of complications can be significantly reduced, leading to a decrease in morbidity and mortality [10].

In Karachi, Pakistan, the Baqai Institute of Diabetology and Endocrinology (BIDE), Baqai Medical University (BMU), pioneered the concept of a station-based 24-hour helpline service in 1996. A randomized trial was conducted to determine the effect of helpline service intervention on HbA1c in patients with diabetes from rural areas of Pakistan, which showed a significant improvement in HbA1c levels [9]. To the best of our knowledge, no such records were found for managing diabetes during Ramadan at the national and international levels. Therefore, the aim of our study is to assess the role of the 24-hour helpline service in the management of diabetes during the holy month of Ramadan.

## Materials and methods

This prospective study was conducted at BIDE-BMU, a tertiary care diabetes unit in Karachi, Pakistan. The duration of the study was from December 2017 to August 2018. Ethical approval for the study was obtained by the Institutional Review Board (IRB) of BIDE. Informed consent was obtained from each study participant. Patients with type 1 and type 2 diabetes who were willing to observe the fast in Ramadan 2018 were included. Patients with poorly controlled type 1 and type 2 diabetes, a history of diabetic ketoacidosis in the last two months, pregnant women with diabetes, and those who were not willing to observe the fast were excluded from the study.

Baseline demographic parameters (gender, age, marital status, smoking habit, duration of diabetes, family history of diabetes) and anthropometric measurements (body mass index (BMI) and systolic and diastolic blood pressure) were obtained. Details of biochemical parameters, including the lipid profile, serum creatinine, urine D/R (protein), and glycated hemoglobin A1c of pre- and post-Ramadan were also recorded. Pre-Ramadan diabetes education related to blood glucose targets, self-monitoring blood glucose (SMBG), nutrition, medication regimens, insulin handling, hypoglycemia, hyperglycemia, alteration of insulin dose, diet changes during Ramadan. Diabetes self-management education on a one-to-one basis was given to each patient. The 24-hour helpline service has been functioning at BIDE since 1996 and is facilitating its registered patients through qualified and trained diabetes educators. Each participant was provided the 24-hour helpline number for queries and support regarding their medical conditions. SMBG recording books were provided to study participants, and they were asked to record their blood glucose level before the pre-dawn meal (suhoor), two hours after suhoor, at mid-day, before the pre-sunset meal (iftar), two hours after iftar, before sleeping, and at any time when the symptoms of hypoglycemia/hyperglycemia or acute illnesses occur during Ramadan. Each patient was given a call by the helpline service (every fifth day of Ramadan) and SMBG records were noted. Calls were also received from patients for their arising complaints and were facilitated with appropriate advice. All data were collected through the health management system (HMS) of BIDE.

Patients were guided to break the fast, if they felt unwell, or if their blood glucose reached <70 mg/dL (3.9 mmol/L) or >300 mg/dL (16.7 mmol/L) [11]. Glycemic control was assessed by checking HbA1c one month before the start of Ramadan and one month after the end of Ramadan using the high-performance liquid chromatography (HPLC) method on Bio-Rad D-10 (Bio-Rad Laboratories, Inc., Hercules, CA). To determine triglycerides, the glucose oxidase-phenol and phenol + aminophenazone (GOD-PAP) method on a fully automated analyzer was used. Serum total cholesterol was analyzed by the cholesterol oxidase and phenol + aminophenazone (CHOD-PAP) method on Selectra Pro S (ELITech Group SAS, Puteaux • France), a fully automated analyzer. The homogeneous enzymatic colorimetric method was used for high-density lipoprotein (HDL) cholesterol and the direct method used for low-density lipoprotein (LDL) cholesterol measurement. Nephropathy was defined as protein > 1+ on dipstick (Combur 10, Roche Diagnostics, Basel, Switzerland) with no other abnormal findings on urinary examination [12]. Height was measured to the nearest 0.1 cm, with the subject standing in the erect posture, and weight was measured with a portable weighing scale to the nearest 0.1 kg. Body mass index (BMI) was measured as the ratio of weight (kg) to height squared (m2). As per Asian guidelines, people having BMI ≥ 25 (kg/m2) were labeled obese [13]. The blood pressure of the participants was monitored by the mercury sphygmomanometer in a sitting position by using the standard method. Hypertension was defined as blood pressure ≥140/90mmHg [11].

Statistical analysis

Data were analyzed using Statistical Package for Social Sciences (SPSS), version 20.0 (IBM Corp. Armonk, NY). The student's t-test, chi-square test, paired t-test, and two proportion z-test were used where applicable. Continuous variables were presented as mean ± SD. Similarly, categorical variables were presented in the form of numbers and percentages. P-value <0.05 was considered statistically significant.

## Results

A total of 102 patients, 41 (40.2%) with type 1 diabetes and 61 (59.8%) with type 2 diabetes were included. The mean age of patients with type 1 diabetes was 17.02±5.22 years and patients with type 2 diabetes was 49.48±9.68years. Most of the patients were male in both groups. The mean BMI of patients with type 1 diabetes was 22.1±3.6 kg/m2 and type 2 diabetes was 30.37±5.24 kg/m2, and the mean duration of diabetes was 8.39±6.55 years and 10.43±6.53 years, respectively. A family history of diabetes was found in 16 (27.1%) patients with type 1 diabetes and 43 (72.9%) patients with type 2 diabetes. Systolic/diastolic blood pressure was 109.39±14.5/72.8±8.22 in patients with type 1 diabetes and 123.75±14.6/79.67±9.01 in patients with type 2 diabetes. Nephropathy was found in eight (33.3%) patients with type 1 diabetes and in 16 (66.7%)patients with type 2 diabetes. No significant differences were observed for total cholesterol, HDL, LDL, triglyceride, and serum creatinine between both groups (Table 1).

**Table 1:** Baseline demographic and clinical characteristics of patients with type 1 and type 2 diabetes Data presented as mean ± SD or n (%); *p-value < 0.05 was considered statistically significant Student's t-test and chi-square test were applied.

Parameters	Type 1	Type 2
n	41(40.2%)	61(59.8%)
Age (years)	17.02±5.22	49.48±9.68*
Gender		
Male	29(41.4%)	41(58.6%)
Female	12(38.7%)	19(61.3%)
Body mass index (kg/m^2^)	22.1±3.6	30.37±5.24*
Marital status		
Single	33(91.7%)	3(8.3%)*
Married	8(12.3%)	57(87.7%)*
Smoking habit		
No	41(41.4%)	58(58.6%)
Yes	0(0%)	2(100%)
Duration of diabetes (years)	8.39±6.55	10.43±6.53
Family history of diabetes		
No	25(61%)	16(39%)
Yes	16(27.1%)	43(72.9%)*
Systolic blood pressure (mmHg)	109.39±14.5	123.75±14.6
Diastolic blood pressure (mmHg)	72.8±8.22	79.67±9.01
Nephropathy		
No	11(33.3%)	22(66.7%)
Yes	8(33.3%)	16(66.7%)
High density lipoprotein cholesterol (mg/dl)	34.25±8.24	35.84±8.62
Low density lipoprotein cholesterol (mg/dl)	92.06±36.44	88.4±43.97
Total cholesterol (mg/dl)	154.56±33.19	155.11±50.61
Triglyceride (mg/dl)	118.5±52.86	190.38±91.14
Serum creatinine (mg/dl)	1±0.34	1.1±0.21

Table 2 shows the reasons for the calls during Ramadan by type 1 and type 2 patients. Twelve (2.27%) type 1 patients and 41 (10.64%) type 2 patients called because of hypoglycemia. Ninety-four (17.83%) type 1 patients and 31 (8.05%) type 2 patients called due to hyperglycemia. One-hundred twenty-seven (24.09%) type 1 patients and 79 (20.51%) type 2 patients called for an adjustment in their insulin dose because of uncontrolled glucose levels. One-hundred sixty-two calls were received from patients, 85 (16.12%) from type 1 and 77 (20%) from type 2, requesting advice about various concerns, including learning of insulin techniques, information about insulin handling, oral medication inquiry, diet inquiry, timings of insulin, hypoglycemia, and hyperglycemia.

**Table 2:** Frequency of calls and reasons on the basis of type of diabetes Data presented as n (%); p-value<0.05 was considered statistically significant Two-proportion z-test was applied. SMBG: self-monitoring blood glucose

Reason for calls	Type 1 diabetes	Type 2 diabetes	p-value	Overall
Number of calls	527	385	-	912
Hypoglycemia	12(2.27%)	41(10.64%)	<0.0001	53(5.81%)
Hyperglycemia	94(17.83%)	31(8.05%)	0.001	125(13.71%)
Insulin dose adjustment	127(24.09%)	79(20.51%)	0.281	206(22.59%)
SMBG details	209(39.65%)	157(40.77%)	0.327	366(40.13%)
Others	85(16.12%)	77(20%)	0.17	162(17.76%)

During the study, 11 patients were asked to break the fast due to symptoms of hypoglycemia and hyperglycemia according to the International Diabetes Federation in collaboration with the Diabetes and Ramadan International Alliance (IDF-DAR) practical guidelines and diabetes and Ramadan practical guidelines [11,14]. Among them, four experienced hypoglycemia and seven developed hyperglycemia. An extra dose was advised to 13 patients 25 times. No patient developed diabetic ketoacidosis (DKA), severe hypoglycemia, or other emergency condition during Ramadan.

The mean HbA1c (%) level before and after Ramadan was 8.94±1.72 and 8.70±2.03 in patients with type 1 diabetes and 8.59±1.73 and 7.71±1.27 in patients with type 2 diabetes, respectively (Table 3).

**Table 3:** Comparison of pre-and post-Ramadan HbA1c of patients with type 1 and type 2 diabetes. Data presented as mean ± SD; P-value < 0.05 was considered as statistically significant. Paired t-test was applied. HbA1c: glycated hemoglobin

Type of DM	HbA1c level before Ramadan	HbA1c level after Ramadan	Mean difference	P-value
Type 1	8.94±1.72	8.70±2.03	0.24	0.181
Type 2	8.59±1.73	7.71±1.27	0.88	0.005
Overall	8.77±1.72	8.21±1.75	0.56	0.002

**Figure 1:**
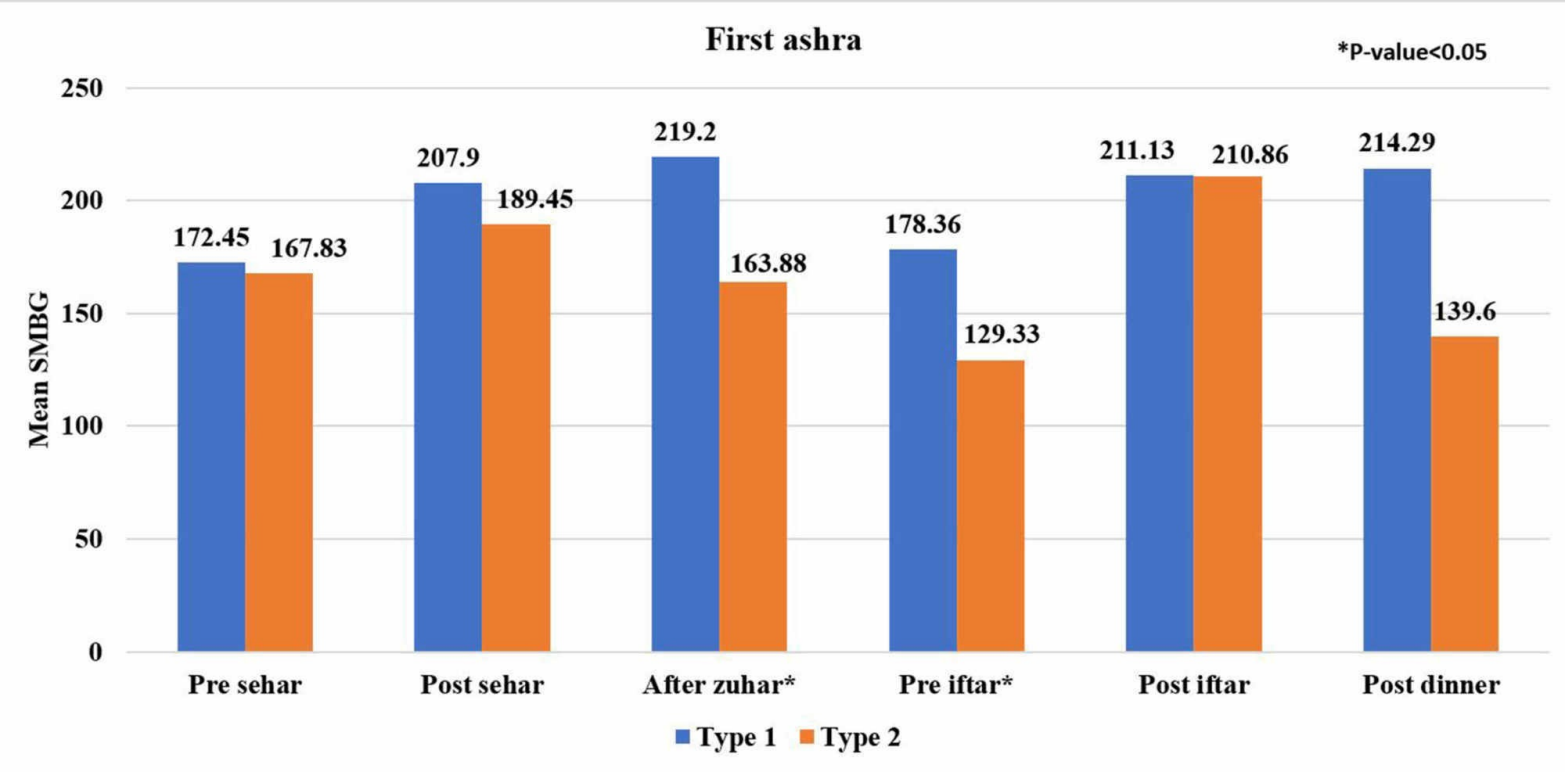
Comparison of self-monitoring blood glucose (SMBG) readings between type 1 and type 2 diabetic subjects in the first ashra

**Figure 2:**
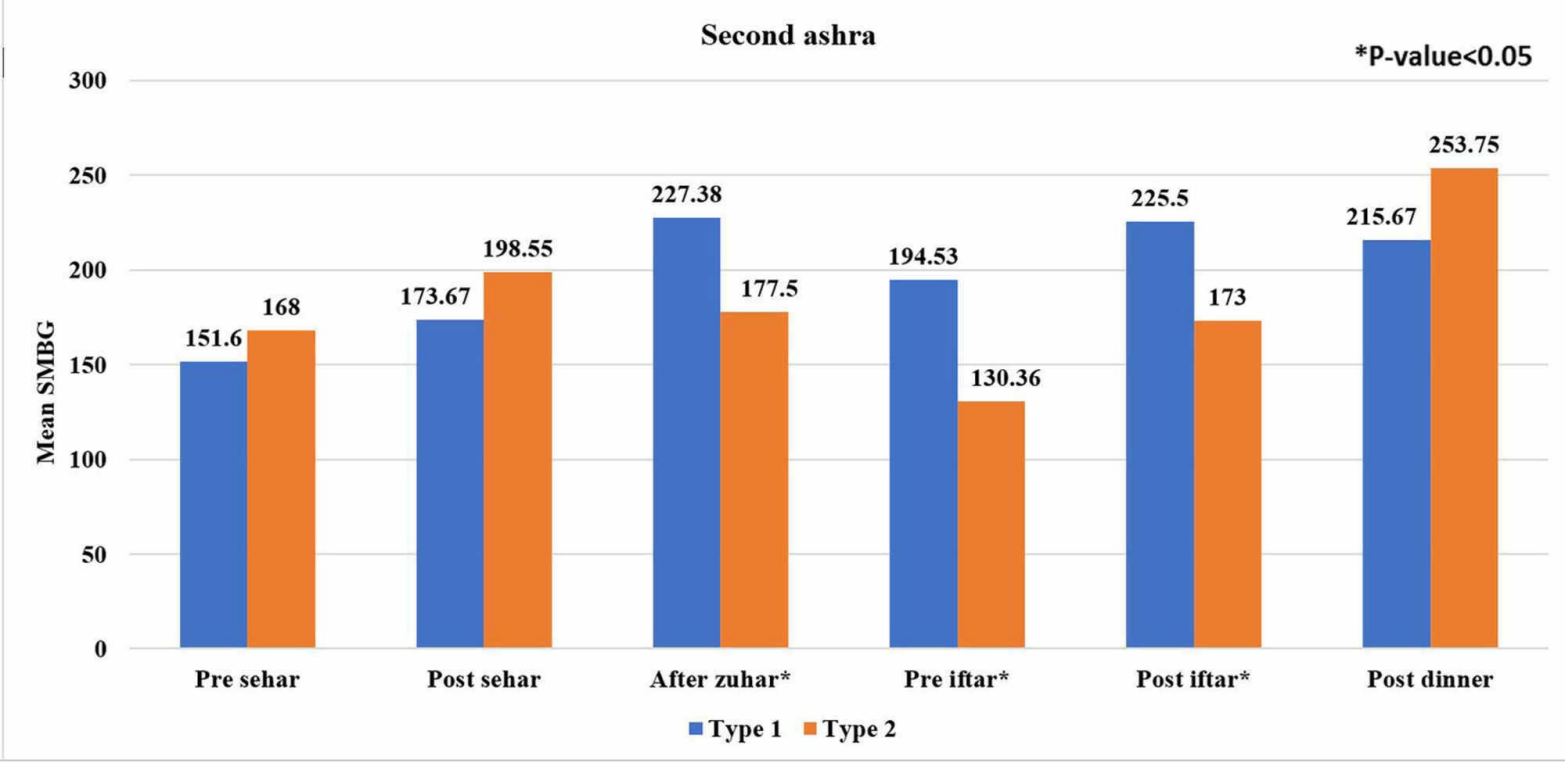
Comparison of self-monitoring blood glucose (SMBG) readings between type 1 and type 2 diabetic subjects in the second ashra

**Figure 3:**
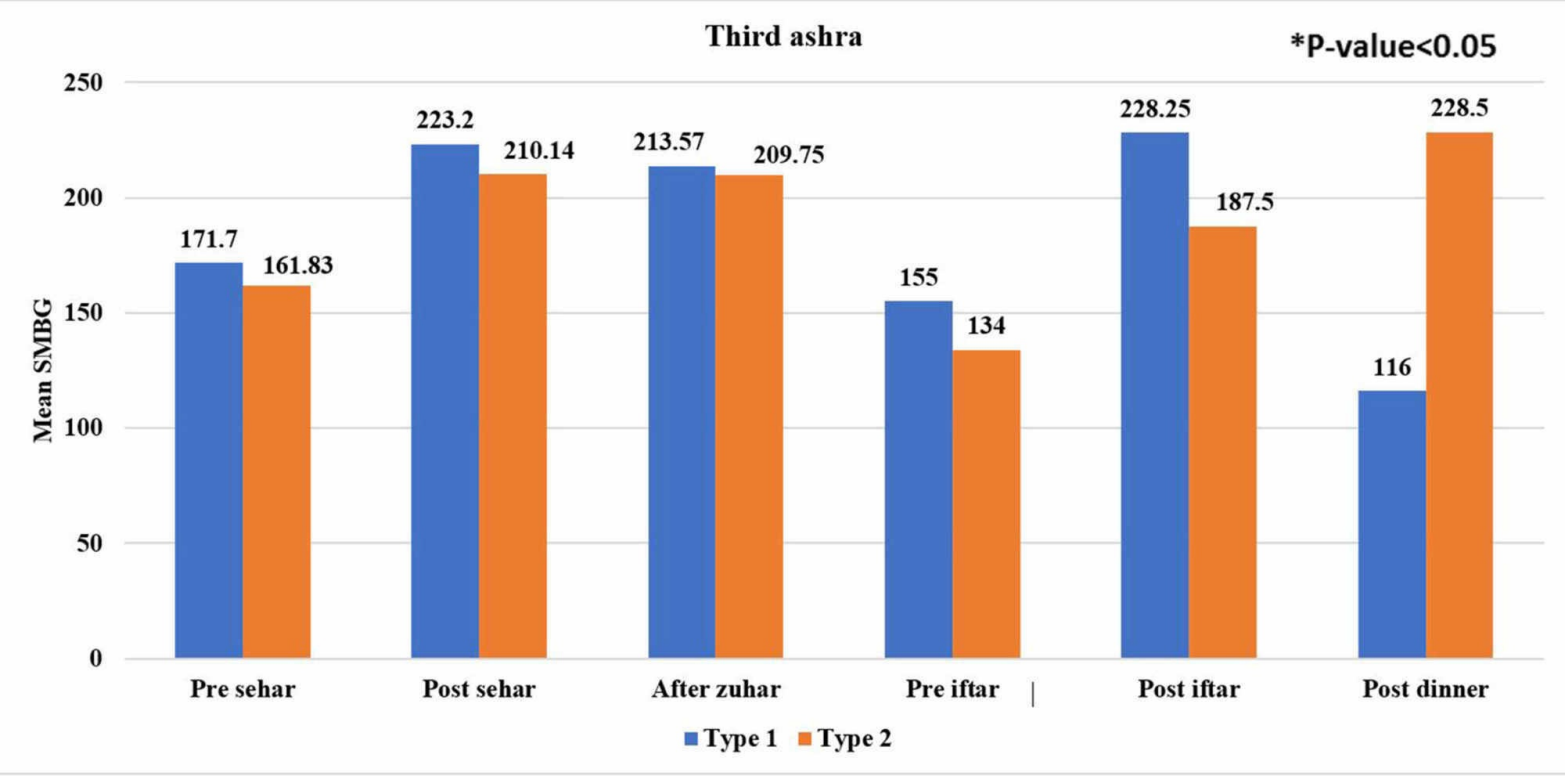
Comparison of self-monitoring blood glucose (SMBG) readings between type 1 and type 2 diabetic subjects in the third ashra

Figure 1, Figure 2, and Figure 3 show the trends of mean SMBG readings during the first 10 days, the second 10 days, and the last 10 days of Ramadan at different meal timings between both groups. Blood glucose level readings were noted by most of the patients (96%) during Ramadan. In the last 10 days of Ramadan, decreasing trends of mean SMBG in patients with type 1 diabetes were observed for pre-Seher, pre-Iftar, and post-dinner while this trend in type 2 diabetes was found for pre-Seher and post-Iftar.

**Figure 4:**
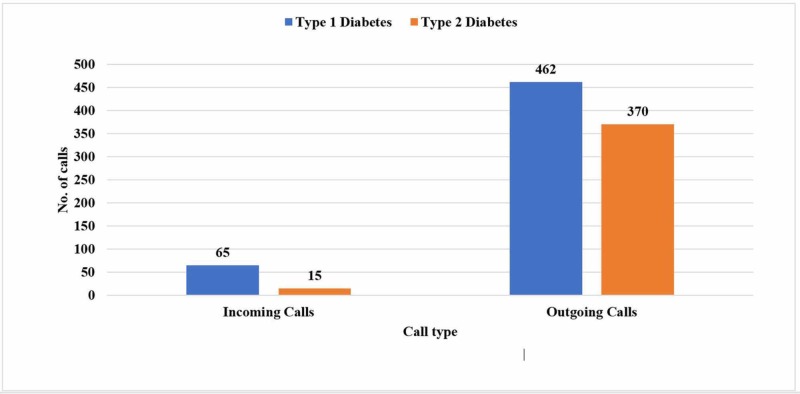
Frequency of calls on the basis of the type of diabetes

Figure 4 shows the frequency of incoming and outgoing calls to both types of diabetics during Ramadan. A total of 912 calls was made to patients using the 24-hour helpline service. Out of which, type 1 calls were 527 (57.7%) - 462 outgoing calls and 65 incoming calls. Type 2 calls were 385 (42.21%) - a total of 370 outgoing calls and 15 incoming calls.

## Discussion

Overall, the mean HbA1c level after Ramadan was significantly decreased in patients with type 2 diabetes whereas it remained the same in patients with type 1 diabetes. It shows that the impact of the 24-hours helpline service and pre-Ramadan education was similar to a previous study for people with diabetes [9]. In Ahmedani MY et al.'s study, patients with pre-Ramadan diabetes education also showed significantly better results for Ramadan-specific diabetes management recommendations as compared to patients without diabetes education [15].

The findings of our study indicate that fasting itself decreases the blood sugar level if patients were given prompt management advice prior to Ramadan. A number of studies reported that the helpline service facilitates regular treatment advice and support and proved to be one of the better tools to deliver regular alerts and reminders to achieve the desired goals [16-19]. To the best of our knowledge, at the national and international levels, no such records related to the 24-hour helpline services during Ramadan were observed; reporting this data is the strength of our study. By following Ramadan and diabetes management guidelines, patients were properly guided for extra doses of insulin adjustment and diet by the24-hour helpline service and no patient throughout the study was admitted with DKA or severe hypoglycemia/hyperglycemia to the emergency department, which shows the effectiveness of the 24-hour helpline service in Ramadan.

In this study, most of the patients (96%) in Ramadan noted their blood glucose level similar to the Ahmedani MY et al. study, which is due to pre-Ramadan diabetes education to rectify the misconception that checking blood glucose during fasting can break the fast [20]. It was also due to proper follow-up by our diabetes educators that they made a call every fifth day of Ramadan to each patient to record the SMBG. Diabetes education prior to Ramadan and SMBG played a key role in managing diabetes, minimizing glucose variability, helping predict severe hypoglycemia, and achieving HbA1c targets similar to the Schnell O et al. study [21]. Our results, similar to the Noon MJ et al. study, indicate that people with diabetes, having no complications, and willing to fast, can fast if they are provided pre-Ramadan education and support was delivered through a helpline service during Ramadan [22].

## Conclusions

Overall, pre-Ramadan diabetes education and treatment adjustment by using a station-based 24-hour helpline service during the holy month of Ramadan has a significant role in controlling blood sugar levels, hence minimizing the complications of diabetes, facilitating timely treatment adjustments, and reducing unnecessary hospital visits and admissions.

## References

[1] (2018). World Health Organization. Global report on diabetes. http://apps.who.int/iris/bitstream/10665/204871/1/9789241565257_eng.pdf.

[2] (2018). International Diabetes Federation. IDF Diabetes Atlas 8th Edition. http://www.idf.org/e-library/...research/...atlas/134-idf-diabetes-atlas-8th-edition.html.

[3] Basit A, Fawwad A, Qureshi H, Shera AS (2018). Prevalence of diabetes, pre-diabetes and associated risk factors: second National Diabetes Survey of Pakistan (NDSP), 2016-2017. BMJ Open.

[4] Chamsi-Pasha M, Chamsi-Pasha H (2016). The cardiac patient in Ramadan. Avicenna J Med.

[5] Al-Arouj M, Assaad-Khalil S, Buse J (2010). Recommendations for management of diabetes during Ramadan. Diabetes Care.

[6] Abolaban H, Al-Moujahed A (2017). Muslim patients in Ramadan: a review for primary care physicians. Avicenna J Med.

[7] Ahmad J, Pathan MF, Jaleel MA (2012). Diabetic emergencies including hypoglycemia during Ramadan. Indian J Endocr Metab.

[8] Abejew AA, Belay AZ, Kerie MW (2015). Diabetic complications among adult diabetic patients of a tertiary hospital in Northeast Ethiopia. Advances in Public Health.

[9] Ahmed F, Asim-Bin-Zafar MR, Ghafoor E, Rehman RA, Uddin Q (2017). Impact of 24 hours helpline service for people with diabetes. Pak J Med Sci.

[10] Diabetes UK Helpline (2018). Diabetes UK helpline. http://www.diabetes.org.uk/careline.

[11] Hassanein M, Al-Arouj M, Hamdy O (2017). Diabetes and Ramadan: practical guidelines. Diabetes Res Clin Pract.

[12] Abro M, Zafar AB, Fawwad A, Mustafa N, Tahir B, Riaz M, Basit A (2018). Prevalence of diabetic micro vascular complications at a tertiary care unit of Karachi, Pakistan. Int J Diabetes Dev Ctries.

[13] (2018). WHO. The Asian-Pacific perspective: redefining obesity and its treatment. https://apps.who.int/iris/handle/10665/206936.

[14] (2018). International Diabetes Federation. Diabetes and Ramadan - practical guidelines. https://www.idf.org/e-library/guidelines/87-diabetes-and-ramadan-practical-2.

[15] Ahmedani MY, Ahsan S, Ul Haque MS (2017). Role of Ramadan specific diabetes education (RSDE); a prospective study. Pak J Med Sci.

[16] Hilliard ME, Sparling KM, Hitchcock J, Oser K, T T, Hood K (2015). The emerging diabetes online community. Curr Diabetes Rev.

[17] Ahmedani MY, Alvi SF, Fawwad A, Basit A (2014). Implementation of Ramadan-specific diabetes management recommendations: a multi-centered prospective study from Pakistan. J Diabetes Metab Disord.

[18] Hui E, Bravis V, Hassanein M (2010). Management of people with diabetes wanting to fast during Ramadan. BMJ.

[19] Benaji B, Mounib N, Roky R (2006). Diabetes and Ramadan: review of the literature. Diabetes Res Clin Pract.

[20] Hassanein M, Al-Arouj M, Hamdy O (2017). Diabetes and Ramadan: practical guidelines. Diabetes Res Clin Pract.

[21] Schnell O, Hanefeld M, Monnier L (2014). Self-monitoring of blood glucose a prerequisite for diabetes management in outcome trials. J Diabetes Sci Technol.

[22] Noon MJ, Khawaja HA, Ishtiaq O (2016). Fasting with diabetes: a prospective observational study. BMJ Glob Health.

